# The cGAS/STING/TBK1/IRF3 innate immunity pathway maintains chromosomal stability through regulation of p21 levels

**DOI:** 10.1038/s12276-020-0416-y

**Published:** 2020-04-13

**Authors:** Abdul Basit, Min-Guk Cho, Eui-Yun Kim, Dohyeong Kwon, Suk-Jo Kang, Jae-Ho Lee

**Affiliations:** 1 Department of Biochemistry and Molecular Biology, Suwon, 443-721 South Korea; 2 Genomic Instability Research Center, Ajou University School of Medicine, Suwon, 443-721 South Korea; 3 Department of Biomedical Sciences, The Graduate School, Ajou University, Suwon, 443-721 South Korea; 4 Department of Biological Sciences, Korea Advanced Institute of Science and Technology, Daejeon, 34141 South Korea; 5 Present Address: Department of Cell Biology, The University of Miami Miller School of Medicine, University of Miami, Miami, FL 33136 USA; 6 Present Address: Medytox Gwangkyo R&D Center, Medytox, Inc., Suwon, 16506 South Korea

**Keywords:** Chromosome segregation, Checkpoints, Interferons, Mitosis, RIG-I-like receptors

## Abstract

Chromosomal instability (CIN) in cancer cells has been reported to activate the cGAS–STING innate immunity pathway via micronuclei formation, thus affecting tumor immunity and tumor progression. However, adverse effects of the cGAS/STING pathway as they relate to CIN have not yet been investigated. We addressed this issue using knockdown and add-back approaches to analyze each component of the cGAS/STING/TBK1/IRF3 pathway, and we monitored the extent of CIN by measuring micronuclei formation after release from nocodazole-induced mitotic arrest. Interestingly, knockdown of cGAS (cyclic GMP-AMP synthase) along with induction of mitotic arrest in HeLa and U2OS cancer cells clearly resulted in increased micronuclei formation and chromosome missegregation. Knockdown of STING (stimulator of interferon genes), TBK1 (TANK-binding kinase-1), or IRF3 (interferon regulatory factor-3) also resulted in increased micronuclei formation. Moreover, transfection with cGAMP, the product of cGAS enzymatic activity, as well as add-back of cGAS WT (but not catalytic-dead mutant cGAS), or WT or constitutively active STING (but not an inactive STING mutant) rescued the micronuclei phenotype, demonstrating that all components of the cGAS/STING/TBK1/IRF3 pathway play a role in preventing CIN. Moreover, p21 levels were decreased in cGAS-, STING-, TBK1-, and IRF3-knockdown cells, which was accompanied by the precocious G2/M transition of cells and the enhanced micronuclei phenotype. Overexpression of p21 or inhibition of CDK1 in cGAS-depleted cells reduced micronuclei formation and abrogated the precocious G2/M transition, indicating that the decrease in p21 and the subsequent precocious G2/M transition is the main mechanism underlying the induction of CIN through disruption of cGAS/STING signaling.

## Cancer: Keeping chromosomes stable during cell division

Signaling through cGAS, a protein that detects DNA in the cytosol, prevents chromosome instability (CIN) in cancer cells by regulating the levels of the cell-cycle inhibitor p21. Alterations in chromosome number or structure are hallmarks of cancer cells, but their contribution to disease is unclear. Previous studies have shown that CIN activates cGAS, triggering the activation of an immune response and cell death. However, Jae-Ho Lee at Ajou University, Suwon, South Korea and colleagues now show that defects in cGAS signaling in cells treated with a drug that stops cell-cycle progression leads to CIN and the formation of extra-nuclear bodies containing damaged chromosome fragments, known as micronuclei. Restoring cGAS activity or increasing p21 expression levels prevented micronuclei formation, highlighting a mechanism through which cancer cells can maintain chromosomal stability.

## Introduction

Innate immunity provides a line of defense against invading pathogens because it detects pathogen-associated molecular patterns (PAMPs) and induces an immune response that eradicates the pathogens. Sometimes, the immune system is activated in the absence of infection owing to the presence of damage-associated molecular patterns (DAMPs) that can be released during sterile inflammation or injury. Accordingly, each cell has various pattern-recognition receptors (PRRs), each of which has a predefined role^1^. Cyclic GMP-AMP synthase (cGAS) is one such PRR that detects cytosolic double-stranded DNA (dsDNA), whether foreign or self. Upon detection of dsDNA, cGAS binds it and synthesizes the second messenger cyclic GMP-AMP (cGAMP)^2,3^. cGAMP then binds the endoplasmic reticulum transmembrane protein stimulator of interferon genes (STING), which becomes active and translocates to the intermediate compartments between the endoplasmic reticulum and Golgi^4^. During translocation, cGAMP recruits TANK-binding kinase-1 (TBK1), which phosphorylates STING, leading to recruitment of interferon regulatory factor-3 (IRF3)^5^. TBK1 phosphorylates IRF3, causing it to dimerize and move into the nucleus, where it induces transcription of genes encoding various cytokines, interferons, and chemokines. TBK1 also phosphorylates Iκβα, an inhibitor of the transcription factor NF-κB (nuclear factor kappa-light-chain-enhancer of activated B cells), marking it for proteasomal degradation; Iκβα degradation releases NF-κB, which translocates together with IRF3 into the nucleus, providing a synergistic response against invading pathogens^6^.

Genomic instability is a hallmark of cancer. The most common causes of genomic instability are chromosomal missegregation and impaired DNA damage repair (DDR) pathways. There are two possible outcomes after a cell has undergone genomic instability: DNA mutations and/or chromosomal instability (CIN)^7^. CIN can be structural or numerical. Structural CIN results in phenotypic manifestations, such as the formation of micronuclei, binuclei, or multinuclei, whereas numerical CIN gives rise to aneuploidy—an abnormal number of chromosomes^8^. However, the prominent effect in chromosomally unstable cells is an increase in the formation of micronuclei, reflecting the fact that this outcome may arise from two major chromosomal segregation errors—lagging chromosome or chromatin bridge formation—during the preceding mitosis. Because cancer cells are known to rapidly proliferate and have compromised cell-cycle checkpoints, they frequently undergo chromosomal missegregation events during mitosis that, upon successive rounds of cell division, result in CIN^8^.

It was previously reported that cGAS is capable of detecting dsDNA inside ruptured micronuclei, which have fragile envelopes; this detection results in the activation of downstream signaling, indicating that CIN activates the cGAS/STING pathway mainly through micronuclei formation^9–13^. The outcome of activation of the cGAS/STING pathway with respect to cancer progression is a matter of controversy. A recent report indicated that activation of this pathway elicits an antitumor response that is subsequently exploited by cancer cells to evade immune surveillance by containing the immune response within the tumor microenvironment at suboptimal levels and promoting tumor metastasis through activation of the noncanonical NF-κB pathway^14^. However, some reports have suggested an opposite role of cGAS/STING pathway activation in tumor progression and metastasis, suggesting that cancer cells with elevated levels of cGAS/STING/IRF3 proteins show enhanced cGAS–STING pathway activation, which induces mitochondrial outer-membrane permeabilization and causes apoptotic cell death^15,16^.

Given that there are numerous reported effects of CIN on cGAS activation via micronuclei formation, the relevant mechanism, namely, the effects of the cGAS/STING pathway on CIN, has not attracted serious research interest. Nevertheless, it has been suggested that cGAS can indirectly decrease CIN by detecting cytosolic DNA in the form of micronuclei and eliciting an innate immune responses that removes cells with CIN. However, whether cGAS can directly contribute to CIN, that is, without the involvement of immune responses, has not been addressed. Here, we demonstrate for the first time that stably decreasing cGAS expression levels in different cancer cell lines (HeLa and U2OS) facing nocodazole-induced mitotic arrest induces chromosomal missegregation events, leading to increased micronuclei formation. Moreover, cGAS add-back or cGAMP transfection rescues micronucleated cells by restoring proper regulation of chromosomal segregation, an effect that is dependent on cGAS enzymatic activity. We also found that the downstream pathway components STING, TBK1, and IRF3 are necessary for the induction of proper chromosomal segregation. Interestingly, our data suggest that these effects of the cGAS/STING/TBK1/IRF3 pathway are mediated by p21 downregulation.

## Materials and methods

### Antibodies

The following antibodies were used: mouse monoclonal antibodies to α-tubulin (Santa Cruz, sc23948), GAPDH (Santa Cruz, sc32233), phospho-H2A.X (Ser139) (EMD Millipore, 05-636), anti-γ-tubulin (Sigma-Aldrich, T5326), and IRF3 (Abcam, ad50772), which were used at a 1:1000 dilution in phosphate-buffered saline (PBS) with 3% bovine serum albumin (BSA); rabbit monoclonal antibodies to cGAS (Cell Signaling, #15102S), STING (Cell Signaling, 13647), phospho-IRF3 (Ser386) (Abcam, ab76433), phospho-IRF3 (Ser396) (Cell Signaling, #29047S), phospho-TBK1 (Ser172) (Cell Signaling, 5483S), and TBK1 (Cell Signaling, 3504S), which were used at a 1:1000 dilution in PBS with 3% BSA. Horseradish-peroxidase-conjugated anti-mouse (G21040) and anti-rabbit (G21234) antibodies were obtained from Invitrogen (at a 1:3000 dilution in TBST). The following fluorochrome-conjugated secondary antibodies were used at a 1:500 dilution in PBS with 3% BSA: anti-mouse Alexa-488 (Invitrogen, A11059), anti-rabbit Alexa-488 (Invitrogen, A11034), and anti-mouse Cy3 (Jackson ImmunoResearch, 715-165-151).

### Cell culture

All cells used herein were purchased from American Type Culture Collection (Manassas, VA, USA) unless otherwise noted, and they were cultured in Dulbecco’s modified Eagle’s medium (DMEM)/high glucose (Gibco, 31600-034) supplemented with penicillin/streptomycin (Gibco BRL, 15240-062) and 10% (V/V) fetal bovine serum (FBS) (Gibco BRL, 16000-044) at 37 °C in a humidified incubator with 5% CO_2_. The hTERT-immortalized retinal pigment epithelial cell line hTERT-RPE1 was obtained from ATCC and was cultured in DMEM/F12 supplemented with penicillin/streptomycin, 10% FBS, and 0.01 mg/mL hygromycin B (Sigma-Aldrich, H0654). The cGAS-deficient HeLa cell line was generated through TALEN-mediated gene disruption^17^.

### Synchronization and drug treatment

To synchronize the cells at the G1/S phase by double thymidine block (DTB), cells were grown on coverslips and incubated in growth medium containing 1 mM thymidine (Sigma, T9250) for 16 h. Cells were then released from the thymidine block by first washing with thymidine-free medium (first release) and then culturing them in growth medium for 8 h. Subsequently, cells were subjected to a second thymidine block for an additional 16 h. For G2/M phase-arrested cells, cells were synchronized by the double thymidine block as described above, and then cells that were previously arrested with thymidine for 16 h were washed with thymidine-free medium before being cultured in complete medium for 7 h (for HeLa cells) or 8 h (for U2OS cells). Then, the cells were cultured in medium containing 9 μM RO3306 (Enzo, ALX-270_463) to arrest cells at the G2/M transition for 2 h (for HeLa cells) or 3 h (for U2OS cells). To induce mitotic arrest, cells were synchronized at prometaphase with 100 ng/mL nocodazole (Sigma, M1404) for 16 h. To inhibit protein degradation, cells were treated with 10 μM MG132 (Sigma, C2211) for 8 h after knocking down STING. For inhibition of protein translation, cells were transfected with siRNA targeting STING and then were treated with 10 μg/mL cycloheximide (Sigma, C7698) for the indicated time intervals.

### Plasmids and transfection experiments

DNA encoding human cGAS and human STING were cloned by PCR and then were subcloned into N-Myc-pcDNA3.1(+)Myc-HisC (Thermo Fisher Scientific) and N-Flag-pcDNA3.1(+)Myc-HisC plasmids, respectively. The plasmid encoding HA-p21 was a gift from Dr. Jaewhan Song (Yonsei University, Korea). RFP-cGAS (plasmid # 86676) was purchased from Addgene. A MYC-cGAS mutant (E225A, D227A), MYC-cGAS mutant (siRNA-resistant), Flag-STING mutant (R284M), and Flag-STING mutant (L374A) were constructed using a Muta-Direct^TM^ site directed mutagenesis kit (iNtRON Biotechnology, #15071). 2′3′-cGAMP was purchased from Invitrogen. HeLa cells were transfected using Neon electroporation (Invitrogen), Lipofectamine® 2000 transfection reagent (Invitrogen), or ViaFect^TM^ transfection reagent (Promega) according to the manufacturers’ protocols.

### Knockdown experiments

High-performance liquid chromatography-purified (>97% pure) small interfering RNA (siRNA) oligonucleotides targeting cGAS, STING, TBK1, and IRF3 were purchased from Thermo Fisher. The sequences of the sense strands of the siRNA oligonucleotides were as follows: cGAS #2, 5′-CGUGAAGAUUUC UGCACCU-3′, 5′-AGGUGCAGAAAUCUUCACG-3′; cGAS #4, 5′-GCAAAAGUUAGGAAGCAAC-3′, 5′-GUUGCUUCCUAACUUUUGC-3′; STING (3′-UTR targeting), 5′-UCAUAAACUUUGGAUGCUA-3′, 5′-UAGCAUCCAAAGUUUAUGA-3′; TBK1, 5′-GGAGACAACAACAAGACAU-3′, 5′-AUGUCUUGUUGUUGUCUCC-3′; IRF3, 5′-GGAAGACAUUCUGGAUGAG-3′, 5′-CUCAUCCAGAAUGUCUUCC-3′; and CDKN1A, 5′-CAAGGAGUCAGACAUUUUA-3′, 5′-UAAAAUGUCUGACUCCUUG-3′. Cells were transfected with 10 nM cGAS #2, cGAS #4, STING, TBK1, IRF3, CDKN1A (p21), or control siRNA oligonucleotides using Oligofectamine® Transfection Reagent (Invitrogen) according to the manufacturer’s protocol.

### Immunoblotting

Conventional immunoblotting was performed as previously described using the corresponding antibodies. Briefly, cell lysates (30 μg) were resolved by sodium dodecyl sulfate-polyacrylamide gel electrophoresis and were then transferred to polyvinylidene fluoride membranes. After blocking for 1 h at room temperature (RT) with TBS containing 0.1% (V/V) Tween-20 and 5% (W/V) nonfat milk, membranes were incubated with the corresponding primary antibodies at 4 °C, which was followed by washing with TBS containing 0.1% Tween-20 and incubation with a horseradish-peroxidase-conjugated anti-rabbit or anti-mouse IgG (Amersham Biosciences, Piscataway, NJ) for 1 h at RT. Detection was carried out using ECL reagents (Amersham Biosciences) and exposure of the membranes to X-ray film.

### Immunocytochemistry

Mitotic cells were split onto poly-l-lysine (PLL, P6282, Sigma-Aldrich)-coated slides. Then, cells growing on the slides and fixed in 100% methanol for 15 min at −20 °C. Fixed cells were preincubated in blocking solution (3% BSA in PBS), which was followed by incubation with primary antibodies at 4 °C overnight. Cells were then washed three times in PBS with shaking and then were probed with fluorophore (Cy3 or Alexa Fluor 488)-conjugated anti-mouse or anti-rabbit secondary antibodies. After washing three times with PBS, DAPI (Invitrogen, D3571) was used for DNA counterstaining. Three washes with PBS were followed by mounting in mounting solution (Biomeda, M01). The samples were examined under a fluorescence microscope (Axio Imager M1, Carl Zeiss).

### Time-lapse analysis

HeLa cells were transfected with siControl or sicGAS and then were treated with nocodazole (100 ng/mL) for 16 h. Then, cells at prometaphase were collected by shaking the dish, and then they were seeded (1 × 10^4^ cells/well) in a four-well glass dish (Thermo Scientific™ Nunc™Lab-Tek II Chambered Coverglass, MA, USA) and incubated overnight in standard culture conditions to enable estimation of the duration of mitosis along with visualization of unstable chromosomal phenotypes with time-lapse photomicroscopy. To visualize chromosomes, cells were incubated with 1 μg/mL Hoechst 33342 (Thermo Scientific™ Hoechst® 33342, MA, USA) for 30 min. Fluorescence images were acquired every 5 min for 24 h while using a Nikon eclipse Ti camera (Tokyo, Japan) with a ×40 dry Plan-Apochromat objective. Images were captured with an iXonEM +897 Electron Multiplying charge-coupled device camera (Teledyne Princeton Instruments, Trenton, NJ, USA) and analyzed in the Nikon Imaging Software (NIS)-elements advanced research (AR) (Nikon, Tokyo, Japan).

### Mitotic index

Mitotic cells were stained with aceto-orcein solution in 60% acetic acid (Merck, ZC135600) to visualize the condensed chromosomes. To determine the mitotic index, the percentage of mitotic cells with condensed chromosomes was quantified under a light microscope.

### Statistical analysis

Most data are presented as the means ± standard deviations (SDs). Each experiment was performed in triplicate. Statistical differences were analyzed by Student’s *t*-test, and asterisks (*) indicate significant differences: *P < 0.05; **P < 0.01; and ***P < 0.005.

## Results

### CIN is enhanced in cGAS-depleted cells

**Fig. 1:**
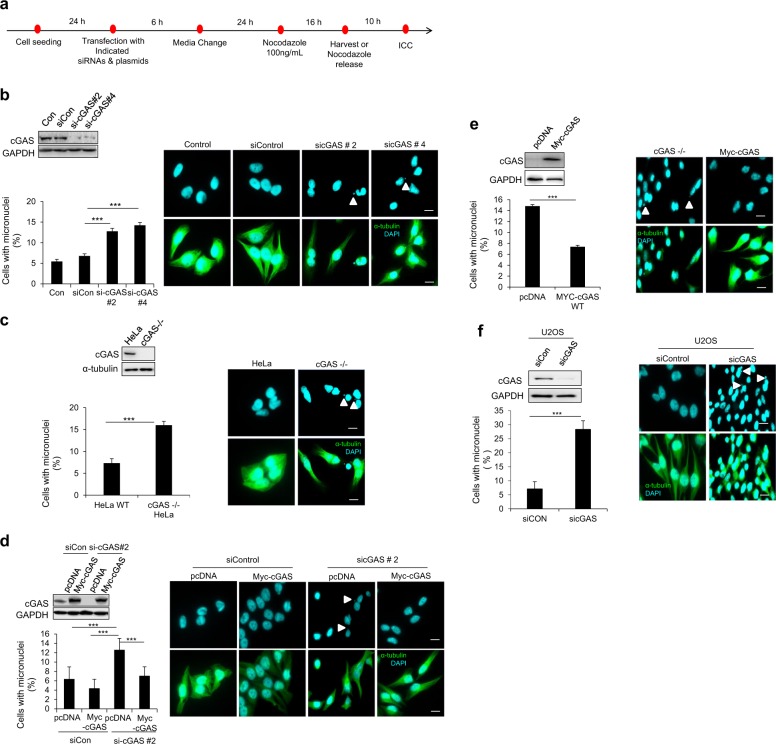
cGAS prevents the formation of micronuclei. **a** Generalized experimental scheme utilized to identify the role of the cGAS–STING pathway in the induction of chromosomal instability. **b** Thirty hours after transfection with the cGAS siRNA, nocodazole treatment (100 ng/mL) commenced for 16 h. The mitotic cell fraction was obtained by shaking the plate, and the cells were reseeded on a coverslip; the remaining cells were subjected to western blotting with the indicated antibodies (top panel). Quantification of the percentage of micronucleated cells was performed by immunocytochemistry (bottom panel) along with representative fluorescent images showing micronuclei (white arrowhead denotes micronuclei). The results are given as the mean ± SD from three independent experiments (*n* = 300). ***P < 0.001 as assessed by Student’s *t*-test. **c** Wild-type HeLa and cGAS−/− HeLa cells were treated with 100 ng/mL nocodazole for 16 h. Mitotic cells were collected by shaking the plate, and they were then subjected to western blotting (top panel) and immunocytochemical analysis (bottom panel) to quantify micronucleated cells; representative fluorescent images of micronuclei (white arrowhead denotes micronuclei) are shown. The results are given as the mean ± SD from three independent experiments (*n* = 300). ***P < 0.001 as assessed by Student’s *t*-test. **d** Cells were co-transfection of sicGAS with a control vector or MYC-cGAS (non-targeting siRNA) in HeLa cells for 20 h, and then they were arrested at prometaphase by nocodazole treatment for 16 h. Western blotting with the indicated antibodies was performed to confirm cGAS knockdown and cGAS add-back (top panel). Immunocytochemistry was performed to evaluate micronucleated cells 10 h after release from nocodazole-induced mitotic arrest (bottom panel); representative fluorescent images show micronuclei (white arrowhead denotes micronuclei). The results are given as the mean ± SD from three independent experiments (*n* = 300). ***P < 0.001 as assessed by Student’s *t*-test. **e** Transfection of the MYC-cGAS plasmid into cGAS−/− HeLa cells for 12 h before the treatment with nocodazole for 16 h. Mitotic cells were collected, reseeded, and cultured for 10 h to quantify the number of cells containing micronuclei by immunocytochemistry (bottom panel); representative fluorescent images show micronuclei (white arrowhead denotes micronuclei), and their cell lysates were used for western blotting with the indicated antibodies (top panel). The results are given as the mean ± SD from three independent experiments (*n* = 300). ***P < 0.001 as assessed by Student’s *t*-test. **f** U2OS cells were transfected with sicGAS for 30 h after pretreatment with nocodazole for 16 h. Mitotic cells were subjected to western blotting to determine cGAS and GAPDH (loading control) protein levels (top panel) as well as ICC to enumerate the percentage of cells with micronuclei (bottom panel); representative fluorescent images show micronuclei (white arrowhead denotes micronuclei). The results are given as the mean ± SD from three independent experiments (*n* = 300). ***P < 0.001 as assessed by Student’s *t*-test.

To determine whether cGAS directly contributes to CIN without the involvement of the immune system, to find a model suitable for our experimental setting we addressed the cGAS/STING levels in four cell lines: hTERT-RPE1, U2OS (human osteosarcoma cell line), HEK293T (human embryonic kidney cell line), and HeLa cells. Both cGAS and STING were detected in HeLa and U2OS cells but not in hTERT-RPE1 or HEK293T cells (Fig. S1a). Moreover, STING expression levels were very low in U2OS cells compared with that of HeLa cells (Fig. S1a). We then confirmed that cGAS localizes to micronuclei, as previously reported in cGAS-positive cell lines, following treatment of these cell lines with nocodazole (100 ng/mL) for 16 h to induce mitotic arrest; then cells were released from the arrest for 10 h, which enabled us to observe the cells in subsequent interphase (Fig. S1b, c). To confirm that the cGAS/STING/TBK1/IRF3 pathway is intact and functional in HeLa cells, we introduced dsDNA into these cells by transfecting them with an empty pcDNA vector and then assessed cGAS–STING pathway activation by determining phospho-TBK1 and phospho-IRF3 levels (Fig. S1d). The results of this analysis suggested that HeLa cells were a suitable cell line for investigating the contribution of the cGAS/STING pathway to CIN. Although there is a possibility that the cGAS/STING/TBK1/IRF3 pathway can be indirectly affected by the hypertriploid chromosome number in HeLa cells, which is not known, our decision to use the cell line was based solely on cGAS and STING expression levels as detected by western blot for the four tested cell lines (Fig. S1a). We then used an RNA interference (RNAi) approach to knock down cGAS in HeLa cells; we used two different small interfering RNAs (siRNAs) and measured the extent of CIN by monitoring micronuclei formation 10 h after release from nocodazole-induced mitotic arrest (100 ng/mL nocodazole for 16 h) (Fig. 1a). Interestingly, immunocytochemical analysis revealed a marked increase in the fraction of micronucleated cells among cGAS-depleted cells compared with that of control siRNA-transfected cells (Fig. 1b), suggesting that cGAS is necessary to maintain chromosomal stability within cycling cells. To test this hypothesis, we compared micronuclei formation in wild-type (WT) HeLa and cGAS-knockout HeLa cells. cGAS-knockout HeLa cells exhibited an increased CIN phenotype similar to that of cGAS-knockdown HeLa cells, as evidenced by an increase in the percentage of micronucleated cells compared with that of WT HeLa cells (Fig. 1c). Moreover, the addition of WT cGAS (siRNA-resistant) to cGAS-depleted HeLa cells clearly decreased the number of micronucleated cells, confirming cGAS-dependent regulation of chromosomal stability (Fig. 1d). Additionally, transfection of cGAS-knockout HeLa cells with a plasmid encoding WT cGAS also reduced micronuclei formation (Fig. 1e). To exclude the possibility that this is a cell-type specific response, we transfected U2OS cells with an siRNA targeting cGAS. These cells displayed an increase in micronucleation compared to that of the control cells, which is similar to what was observed in cGAS-knockdown HeLa cells (Fig. 1f). These data clearly suggest that cGAS plays a role in maintaining chromosomal stability.

### Chromosomal segregation defects are enhanced by cGAS depletion

**Fig. 2:**
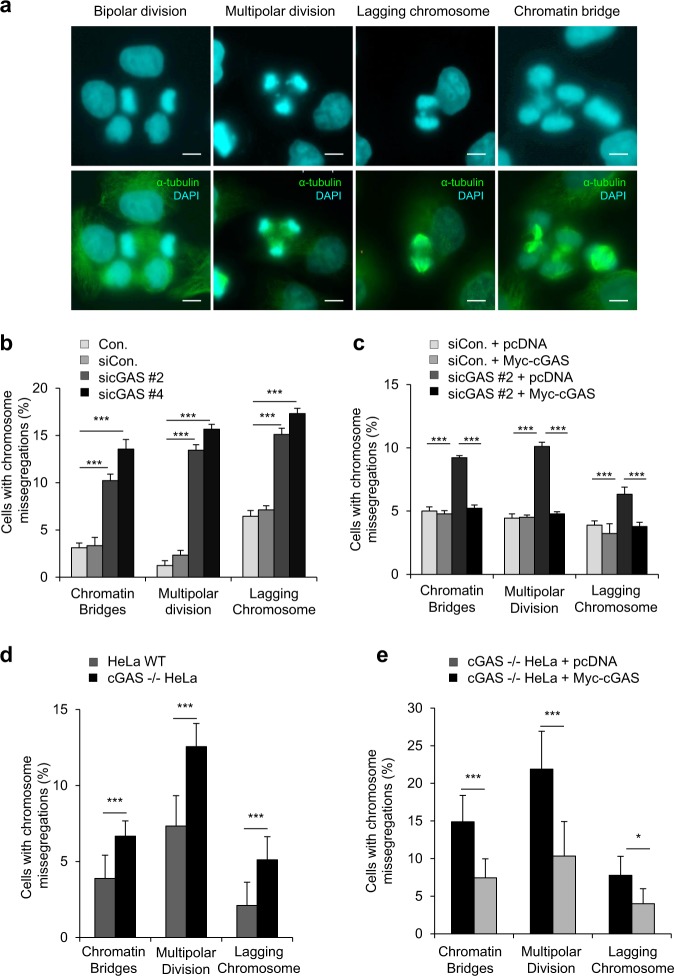
cGAS is essential to regulate proper chromosomal segregations. **a** Representative confocal images of mitotic cells 1 h after release from nocodazole-induced mitotic arrest. Cells showing bipolar division, multipolar division, lagging chromosome, and a chromatin bridge (from left to right). **b** Thirty hours after transfection with sicGAS, mitotic cells were collected by shaking the plate, and they were then treated for 16 h with nocodazole and seeded on a PLL-coated coverslip. After 1 h, cells were fixed with 100% ice-chilled methanol, and ICC was performed. Cells showing the indicated chromosomal segregation errors were calculated as percentages. The results are given as the mean ± SD from three independent experiments (*n* = 300). ***P < 0.001 as assessed by Student’s *t*-test. **c** Co-transfection of sicGAS with pcDNA or MYC-cGAS (non-targeting siRNA) in HeLa cells for 20 h before beginning treatment with nocodazole, which lasted for 16 h. Cells arrested in mitosis were collected and reseeded on PLL-coated coverslips. After 1 h, the percentage of micronucleated cells was quantified using ICC. The results are given as the mean ± SD from three independent experiments (*n* = 300). ***P < 0.001 by Student’s *t*-test. **d** cGAS−/− and wild-type HeLa cells were treated with nocodazole for 16 h. Then, mitotic cells were collected by shaking the plate, and the collected cells were seeded on PLL-coated coverslips for 1 h before ICC was performed to analyze the number of cells showing the indicated chromosomal missegregations. The results are given as the mean ± SD from three independent experiments (*n* = 300). ***P < 0.001 as assessed by Student’s *t*-test. **e** cGAS−/− HeLa cells were transfected with pcDNA or MYC-cGAS and then were treated with nocodazole for 16 h to induce mitotic arrest. After release from nocodazole treatment, mitotic cells were seeded on PLL-coated coverslips for 1 h and were then subjected to ICC to quantify the percentage of cells with chromosomal segregation errors. The results are given as the mean ± SD from three independent experiments (*n* = 300). *P < 0.05, ***P < 0.001 as assessed by Student’s *t*-test.

Micronuclei primarily arise from two basic errors: lagging chromosomes and chromatin bridges, which may be partly induced by multipolar division in the mitotic phase^18^. To address the relationship between these phenotypes and abnormal chromosomal segregation, we monitored cells in anaphase 1 h after release from nocodazole-induced mitotic arrest by counting cells with lagging chromosomes, multipolar division, or chromatin bridges (Fig. 2a). We found that these chromosomal missegregation phenotypes were significantly more abundant in cGAS-knockdown cells than they were in the control cells (Fig. 2b). Add-back experiments in which cGAS-knockdown cells were transfected with an siRNA-resistant WT MYC-cGAS expression plasmid clearly revealed that upon restoration of cGAS protein, cells regained the ability to undergo proper chromosomal segregation (Fig. 2c). Moreover, a comparison of cGAS-knockout and WT HeLa cells undergoing chromosomal segregation 1 h after release from mitotic arrest revealed that more chromosomal segregation errors occurred in cGAS-knockout cells than in WT cells (Fig. 2d). Again, the addition of cGAS to cGAS-knockout HeLa cells rescued the chromosomal missegregation phenotype (Fig. 2e). These findings clearly indicate that cGAS is necessary for normal chromosomal segregation during mitosis, and it suppresses CIN during cell-cycle progression.

### cGAS enzymatic activity is necessary for reducing the micronuclei phenotype

**Fig. 3:**
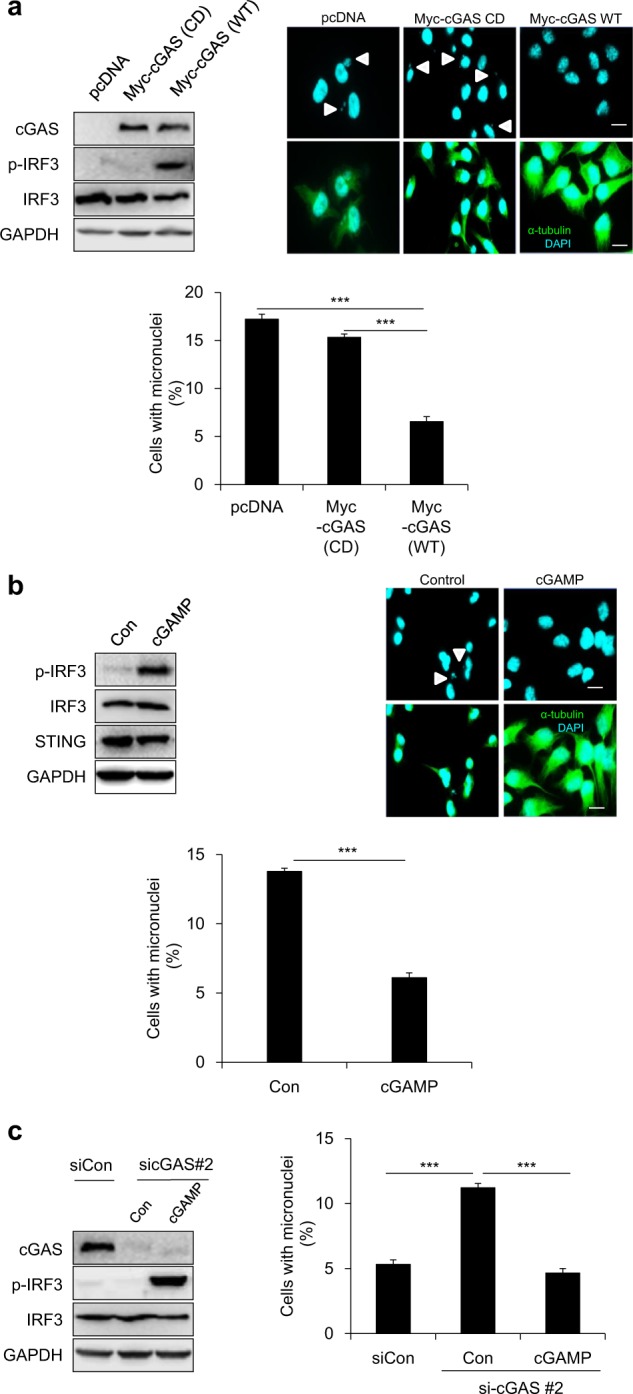
cGAS activity-dependent downregulation of the micronuclei phenotype. **a** cGAS−/− HeLa cells were transfected with the indicated plasmids for 6 h and then were treated with nocodazole for 16 h. Then, mitotic cells were collected by shaking the plate, and they were subjected to western blotting (left panel) with the indicated antibodies. Further, ICC (right panel) was performed to determine the percentage of cells showing micronuclei 10 h after release from nocodazole, and representative fluorescent images show micronuclei (white arrowhead denotes micronuclei). The results are given as the mean ± SD from three independent experiments (*n* = 300). ***P < 0.001 as assessed by Student’s *t*-test. **b** cGAS−/− HeLa cells were transfected with or without cGAMP using Lipofectamine® 2000 transfection reagent and were then incubated for 6 h. Then, the cells were arrested at the M phase via nocodazole treatment for 16 h. A portion of the mitotic cells were seeded on a coverslip to enumerate the cells showing a micronuclei phenotype 10 h after release from arrest, as assessed by ICC (right panel) along with representative fluorescent images showing micronuclei (white arrowhead denotes micronuclei). The remaining portion of cells was subjected to western blotting with the indicated antibodies (left panel). The results are given as the mean ± SD from three independent experiments (*n* = 300). ***P < 0.001 as assessed by Student’s *t*-test. **c** HeLa cells transfected with sicGAS for 20 h were transfected with or without cGAMP for 6 h and then subjected to nocodazole treatment for 16 h. Ten hours after release from nocodazole treatment, western blotting (left panel) with the indicated antibodies and ICC (right panel) was performed to quantify cells displaying a micronuclei phenotype. The results are given as the mean ± SD from three independent experiments (*n* = 300). ***P < 0.001 as assessed by Student’s *t*-test.

Having confirmed the role of cGAS in maintaining chromosomal stability, we sought to determine whether cGAS activity is necessary for this effect. To this end, we transfected cGAS-knockout HeLa cells with plasmids coding WT cGAS or a catalytic-dead (CD) cGAS mutant and then arrested them at mitosis by treating with nocodazole, as described above. Western blotting confirmed that the cGAS CD mutant was unable to induce phosphorylation of IRF3 and thus could not activate the cGAS/STING pathway. Immunocytochemical analysis performed 10 h after release from nocodazole arrest revealed that the cGAS CD mutant, unlike the WT cGAS, was completely incapable of reducing the number of cells containing micronuclei, as summarized in Fig. 3a. Consistent with this, transfection of the cGAS-knockout HeLa cells with cGAMP significantly decreased the number of cells exhibiting micronuclei formation (Fig. 3b). Finally, transfection of cGAS-knockdown HeLa cells with cGAMP also resulted in fewer micronucleated cells than what was observed in the cGAS-knockdown cells transfected with a vehicle control (Fig. 3c). Collectively, these findings conclusively demonstrate that cGAS acts through the promotion of cGAMP synthesis during cell-cycle progression to inhibit micronuclei formation, thus confirming the requirement for cGAS enzymatic activity in reducing the CIN phenotype in successive cell cycles.

### STING acts as a mediator in regulating chromosomal stability

**Fig. 4:**
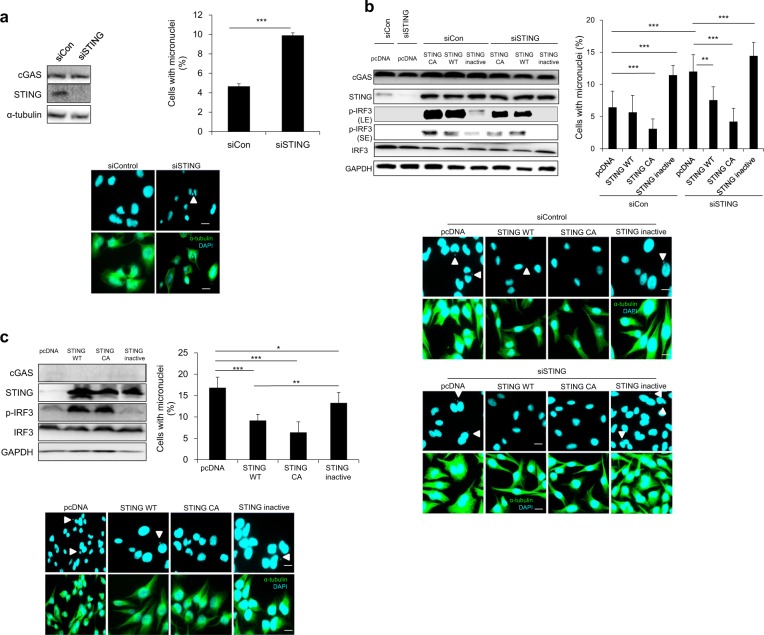
STING activation via cGAS leads to chromosomal stability. **a** HeLa cells transfected with an siRNA targeting STING, and after 30 h they were treated with nocodazole for 16 h. Mitotic cells were subjected to western blotting (left panel) for cGAS, STING, and α-tubulin and to ICC (right panel) to analyze the percentage of micronucleated cells; representative fluorescent images show micronuclei (white arrowhead denotes micronuclei). The results are given as the mean ± SD from three independent experiments (*n* = 300). ***P < 0.001 as assessed by Student’s *t*-test. **b** HeLa cells were cotransfected with siSTING and different indicated plasmids for 20 h and then were arrested via nocodazole treatment. After 16 h, cells were released from arrest, and western blotting (left panel) with the indicated antibodies was performed. The percentages of cells with micronuclei were counted after performing ICC (right panel) on cells 10 h after their release from mitotic arrest; representative fluorescent images show micronuclei (white arrowhead denotes micronuclei). The results are given as the mean ± SD from three independent experiments (*n* = 300). ***P < 0.001 as assessed by Student’s *t*-test. **c** cGAS−/− HeLa cells were transfected with the indicated plasmids for 12 h before nocodazole treatment commenced for 16 h. Ten hours after release from mitotic arrest, cells were subjected to western blotting with the indicated antibodies and ICC to calculate the percentage of cells showing micronuclei formation after nocodazole release; representative fluorescent images show micronuclei (white arrowhead denotes micronuclei). The results are given as the mean ± SD from three independent experiments (*n* = 300). ***P < 0.001 as assessed by Student’s *t*-test.

cGAS activation results in the synthesis of cGAMP, which then binds STING to activate downstream signaling. To determine whether STING plays a role in chromosome stability, we transfected HeLa cells with an siRNA targeting the 3′-UTR of STING (siSTING) and assessed micronuclei formation after release from nocodazole-induced mitotic arrest. Indeed, siRNA-mediated STING knockdown was accompanied by an increase in micronucleated cells compared with that of the siControl-transfected cells, suggesting the requirement of STING in the cGAS-dependent maintenance of proper chromosomal segregation (Fig. 4a). To confirm that this effect from STING is dependent on its activity, we added back WT STING, a constitutively active STING mutant, or an inactive STING mutant to STING-depleted HeLa cells. Western blotting confirmed that both WT and constitutively active STING were able to induce phosphorylation of IRF3, whereas the inactive STING mutant was not. Importantly, the inactive STING mutant failed to abrogate micronuclei formation, whereas cells transfected with either WT STING or constitutively active STING effectively suppressed micronuclei formation (Fig. 4b). We also transfected these mutants into cGAS-knockout HeLa cells and again observed an increase in the percentage of micronucleated cells in cells transfected with either the inactive STING mutant or the control vector; in contrast, transfection of cells with WT STING or constitutively active mutant STING substantially decreased the percentage of micronucleated cells (Fig. 4c). Taken together, these results indicate that STING activation mediates cGAS regulation of chromosomal stability.

### TBK1 and IRF3 are also necessary for the maintenance of chromosomal integrity

**Fig. 5:**
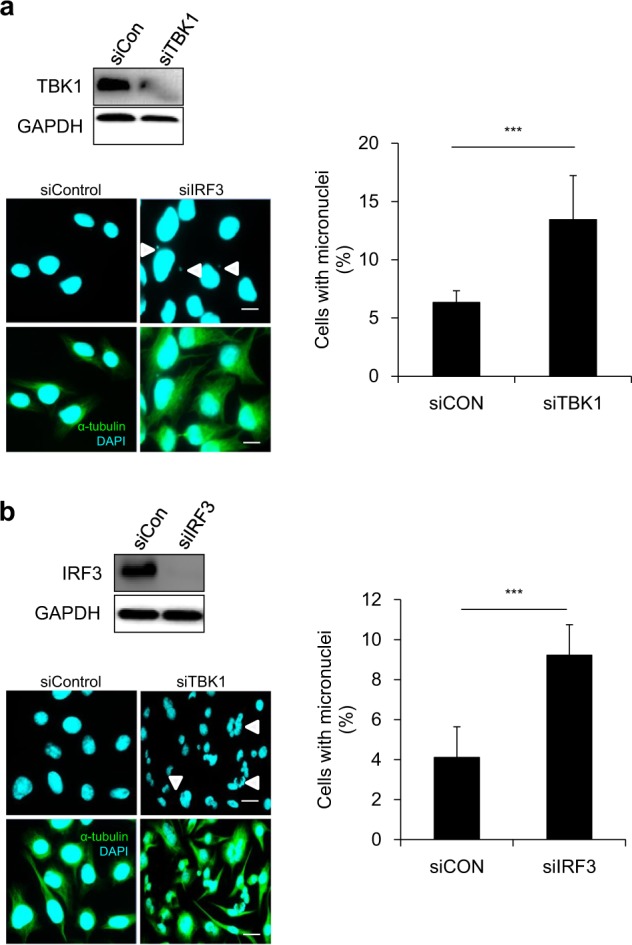
TBK1 and IRF3 downregulation also promotes the formation of micronucleated cells. **a** HeLa cells were transfected with siRNA targeting TBK1, and 30 h later they were treated with nocodazole for 16 h. After 16 h, cells were released from arrest and were subjected to western blotting with the indicated antibodies. Mitotic cells collected after nocodazole treatment were reseeded, and ICC was performed to evaluate the percentage of cells with micronuclei; representative fluorescent images show micronuclei (white arrowhead denotes micronuclei). The results are given as the mean ± SD from three independent experiments (*n* = 300). ***P < 0.001 as assessed by Student’s *t*-test. **b** IRF3 was downregulated by RNA interference in HeLa cells for 30 h, and then cells were treated with nocodazole for 16 h. After 16 h, cells were released from arrest, and mitotic cells were collected and subjected to ICC (right panel) to evaluate the percentage of cells with micronuclei; representative fluorescent images show micronuclei (white arrowhead denotes micronuclei). Western blotting was performed with the indicated antibodies to confirm IRF3 knockdown via siRNA transfection (left panel).

We further tested the possible involvement of downstream components of the cGAS/STING pathway, TBK1 and IRF3, in maintaining chromosomal stability. Knockdown of TBK1 in HeLa cells also resulted in more cells with micronuclei than what was observed following transfection with the siControl (Fig. 5a), suggesting the involvement of TBK1. We then knocked down IRF3 using siRNA and induced mitotic arrest by treatment with nocodazole. Again, cells showing attenuated IRF3 levels displayed increased micronuclei formation compared with that of control cells (Fig. 5b). These data, in addition to our previous findings, indicate that all components of the cGAS/STING/TBK1/IRF3 pathway play discrete roles in maintaining chromosomal stability as cells undergo cell-cycle progression.

### cGAS/STING pathway-dependent expression of p21

**Fig. 6:**
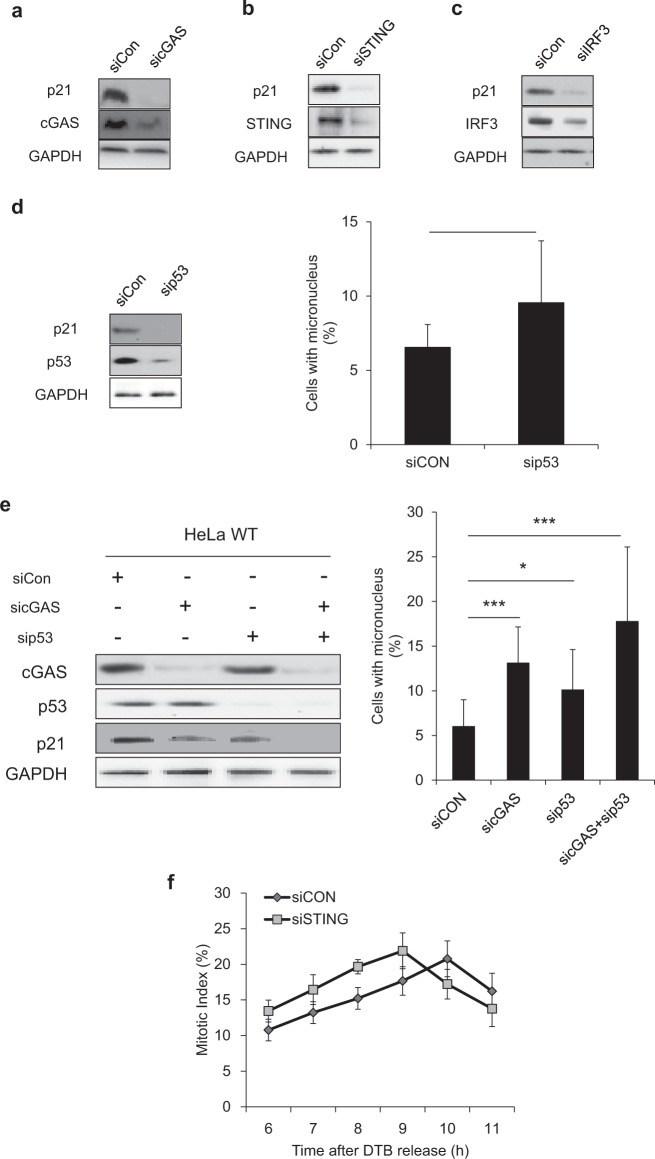
cGAS–STING pathway-dependent p21 expression levels resulting in abnormal cell-cycle progression. **a** HeLa cells were transfected with sicGAS, and after 48 h they were subjected to western blotting with the indicated antibodies. **b** HeLa cells transfected with an siRNA targeting STING, and after 48 h the cell lysates were subjected to western blotting with antibodies against p21, STING, and GAPDH. **c** IRF3 was knocked down using RNA interference, and after 48 h cell lysates were collected. Cell lysates were subjected to western blotting with the indicated antibodies. **d** p53 was knocked down in HeLa cells using sip53, and micronuclei were checked 10 h after release from nocodazole by ICC (right panel); p21 levels were assessed with the indicated antibodies by western blot (left panel). The results are given as the mean ± SD from three independent experiments (*n* = 300). ***P < 0.001 as assessed by Student’s *t*-test. **e** HeLa cells were transfected with sip53, sicGAS, or both, and then micronuclei formation was quantified after release from nocodazole using ICC (right panel). cGAS, p53, and p21 levels were assessed with the indicated antibodies using western blot. The results are given as the mean ± SD from three independent experiments (*n* = 300). ***P < 0.001 as assessed by Student’s *t*-test. **f** HeLa cells were transfected with siSTING and then were subjected to a double thymidine block with 2 mM thymidine for 40 h. Six hours after release from the DTB, quantification of the percentage of mitotic cells with condensed chromosomes was performed by aceto-orcein staining at the indicated time points. The results are given as the mean ± SD from three independent experiments (*n* = 300).

We next asked what the possible mechanism could be by which the cGAS/STING/TBK1/IRF3 pathway regulates chromosomal segregation. This pathway could directly affect the mitotic process or act on the interphase process, which could subsequently induce chromosomal missegregation. Previous reports suggest two possible scenarios in which the cGAS/STING pathway might affect CIN. The first possibility is that it affects centrosome number, given reports that TBK1 interacts with centrosomal proteins to play a role in microtubule stability^19^. However, we found no significant difference in centrosome number after STING depletion or add-back (Fig. S2a, b). The second possibility is that STING acts as a positive regulator of p21 levels through activation of the NF-κB and p53 axes^20^. Since p21 can inhibit cell-cycle progression, including the G2/M transition, downregulation of p21, which is expected in the absence of STING, might affect G2/M transition and subsequent mitotic events as well. Thus, we analyzed the expression levels of p21 protein in cGAS-depleted HeLa cells and found that they were significantly reduced relative to their levels in WT HeLa cells, suggesting that cGAS serves as an upstream signaling protein that regulates p21 levels during cell-cycle progression (Fig. 6a). We further observed that p21 levels were reduced in HeLa cells with STING knocked down (Fig. 6b), as reported previously^20^. We then tested the effects of IRF3 knockdown; previous studies reported that IRF3 enhanced transcriptional activation of p53 and p53-dependent growth inhibition^21,22^. Interestingly, IRF3 downregulation also resulted in significantly decreased p21 levels, suggesting a role for IRF3 in regulating p21 levels in our experimental setting (Fig. 6c). Next, we addressed whether changes in p21 levels by cGAS/STING/TBK1/IRF3 were dependent on p53. We first depleted p53 using siRNA and found that it led to a significant decrease in p21 along with an increase in micronuclei formation, indicating that p21 levels in these cells were dependent on p53 (Fig. 6d). Next, we depleted cGAS, p53, or both and observed that (1) cGAS depletion does not affect p53 levels and (2) even without p53, p21 downregulation was evident after cGAS knockdown, indicating that p21 downregulation in cGAS-depleted cells is p53-independent (Fig. 6e). A reduction in p21 levels would result in precocious mitotic entry, which might induce chromosomal missegregation because of unresolved DNA damage or uncoordinated mitotic entry, among other possibilities^23–25^. Although there was no significant change in the extent of DNA damage in cGAS-depleted cells or cGAMP-transfected HeLa cells (Fig. S2c, d), measurements of the mitotic index after release from the double thymidine block revealed that STING-depleted HeLa cells reproducibly exhibited a G2/M transition that was 1-h earlier than that of WT HeLa cells (Fig. 6d). This suggests the possibility that activation of the cGAS/STING pathway leads to upregulation of p21 during the G2 phase, which would allow cells sufficient time to properly prepare for mitotic entry before entering into mitosis.

### p21 plays an important role in cGAS depletion-induced CIN

**Fig. 7:**
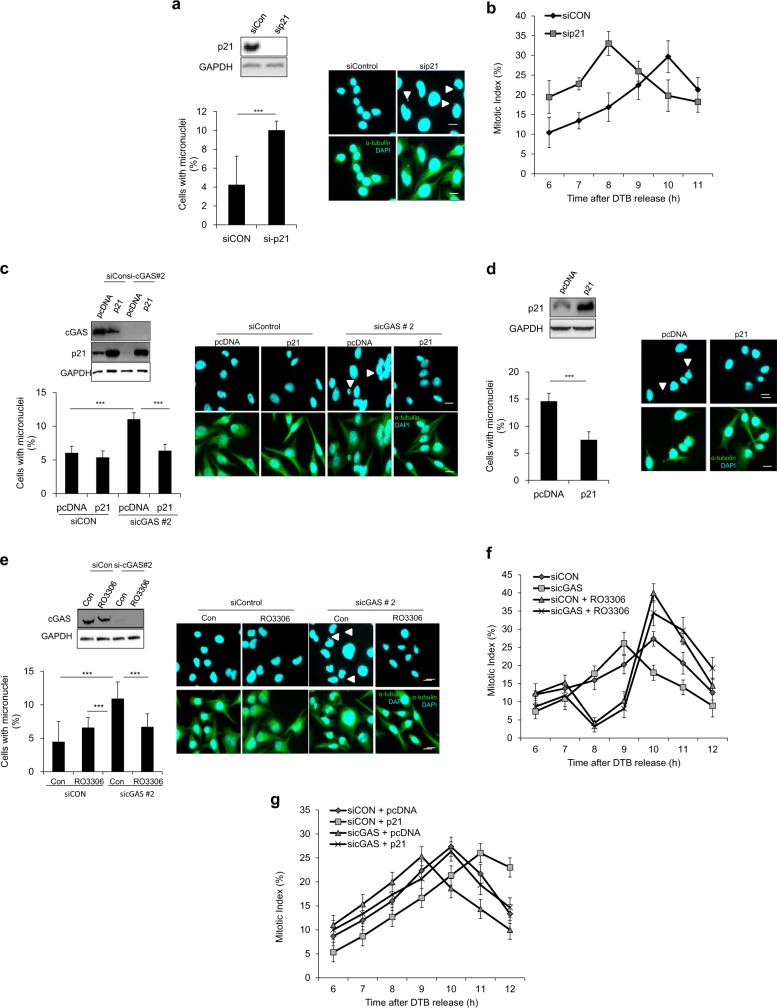
Upregulation of p21 levels or cell-cycle arrest at the G2 phase results in improved chromosomal stability. **a** HeLa cells were transfected with sip21, and after 30 h cells were treated with nocodazole for 16 h. After nocodazole release, cells were subjected to western blotting (top panel) with p21 and GAPDH (loading control) antibodies. Mitotic cells were analyzed by ICC, and the number of micronucleated cells was calculated (bottom panel); representative fluorescent images show micronuclei (white arrowhead denotes micronuclei). The results are given as the mean ± SD from three independent experiments (*n* = 300). ***P < 0.001 as assessed by Student’s *t*-test. **b** Same as in **a**. Mitotic cells (as indicated above) were collected and stained with aceto-orcein to visualize cells with condensed chromosomes. The percentage of mitotic cells with condensed chromosomes was calculated at the indicated time points. The results are given as the mean ± SD from three independent experiments (*n* = 300). **c** Co-transfection of sicGAS with or without a p21 plasmid was performed for 30 h. Then, cells were treated with nocodazole for 16 h before being analyzed by western blot (top panel) with the indicated antibodies as well as by ICC (bottom panel) for enumeration of micronucleated cells; representative fluorescent images show micronuclei (white arrowhead denotes micronuclei). The results are given as the mean ± SD from three independent experiments (*n* = 300). ***P < 0.001 as assessed by Student’s *t*-test. **d** cGAS−/− HeLa cells were transfected with p21 plasmid, and after 12 h they were treated with nocodazole for 16 h. After 16 h, cells were subjected to western blotting (top panel) with the indicated antibodies, and mitotic cells that were collected upon cessation of nocodazole treatment were reseeded on a coverslip so that ICC could be performed. Immunocytochemical analysis (bottom panel) was used to detect and count the percentage of cells showing micronuclei; representative fluorescent images show micronuclei (white arrowhead denotes micronuclei). The results are given as the mean ± SD from three independent experiments (*n* = 300). ***P < 0.001 as assessed by Student’s *t*-test. **e** HeLa cells transfected with sicGAS and synchronized by double thymidine block for 40 h were released from arrest and then 7 h later were treated with RO3306 for 3 h. Mitotic cells with or without RO3306 treatment were collected by shaking the plate at 9 and 10 h, and then they were reseeded on a coverslip to assess chromosomal instability phenotypes, i.e., micronuclei by immunocytochemistry (bottom panel); representative fluorescent images show micronuclei (white arrowhead denotes micronuclei). Western blotting was performed with the indicated antibodies to confirm cGAS downregulation (top panel). **f** Same as indicated in **e**. Six hours after release from DTB, quantification of the percentage of mitotic cells with condensed chromosomes was performed by aceto-orcein staining at the indicated time points; the cells analyzed were those with or without RO3306 treatment for 3 h, which occurred 7 h after release from DTB. The results are given as the mean ± SD from three independent experiments (*n* = 300). **g** Cells were cotransfected with sicGAS and pcDNA (control vector) or p21 and then were subjected to DTB. After release from DTB, cells were harvested at the indicated time points and stained with aceto-orcein to count mitotic cells with condensed chromosomes. The results are given as the mean ± SD from three independent experiments (*n* = 300).

To determine whether the increase in CIN phenotypes (i.e., micronuclei) following attenuation of the cGAS/STING pathway is primarily attributable to a decrease in p21 levels, we downregulated p21 by using an RNAi approach, and we counted the number of cells with micronuclei. Indeed, the percentage of micronucleated cells was robustly increased in p21-deficient cells compared with that of control cells after release from nocodazole-induced mitotic arrest (Fig. 7a); this effect was accompanied by precocious entry into mitosis after release from double thymidine block (Fig. 7b). Next, we tested whether overexpression of p21 could overcome the cGAS-depletion-induced micronuclei phenotype. Importantly, overexpression of p21 in cGAS-knockdown cells resulted in a decrease in the number of cells with CIN compared to that of cGAS-knockdown cells transfected with an empty pcDNA vector control (Fig. 7c); overexpression of p21 also abolished the precocious G2/M transition induced by cGAS depletion (Fig. 7g). Similarly, overexpression of p21 in cGAS-knockout HeLa cells decreased the number of micronucleated cells by approximately 50% compared with that of pcDNA-transfected cGAS-knockdown HeLa cells (Fig. 7d). These data strongly suggest that cGAS depletion-induced CIN due to a decrease in p21 levels following cGAS depletion, which caused precocious entry into mitosis.

If precocious entry into mitosis results in chromosomal missegregation, inducing a delay in G2/M transition under these conditions should decrease CIN phenotypes. To test this hypothesis, we used RO3306, an inhibitor of cyclin-dependent kinase-1 (CDK1), to delay the G2/M transition after release from the double thymidine block and assessed its effect on cGAS depletion-induced CIN. Indeed, RO3306 significantly attenuated cGAS depletion-induced micronuclei formation (Fig. 7e), which is an effect that was accompanied by abrogation of the precocious G2/M transition (Fig. 7f). Collectively, our findings suggest that CIN caused by downregulation of the cGAS/STING pathway arises from a precocious G2/M transition that occurs because of decreased p21 levels.

## Discussion

The cGAS/STING pathway has been extensively studied as a part of the innate immune system. However, reports on its impacts on cell-cycle progression are sparse, and little or nothing is known about its effects on chromosomal segregation. Our findings provide the first evidence for a role for the cGAS/STING pathway in maintaining chromosomal homeostasis as a cell undergoes division. There have been reports of a role for STING in maintaining chromosomal stability via the NF-κB/p53/p21 axis and a role of TBK1 in regulating chromosomal segregation during mitosis through binding to Cep170 and NuMA^19,20^. Another report also suggested that IRF3 overexpression causes cell-cycle arrest at the G1/S phase, resulting in inhibition of DNA synthesis^26^. Some recent reports have suggested that detection of DNA in ruptured micronuclei by cGAS can elicit an immune response that helps eliminate cells with CIN phenotypes, thus indirectly decreasing CIN^15,16^. However, there have been no reports on possible direct contributions of the cGAS/STING/TBK1/IRF3 pathway to CIN. Our findings suggest the first mechanism by which the cGAS–STING pathway directly regulates CIN without involvement of the immune system and demonstrate that all components of the cGAS/STING/TBK1/IRF3 signaling pathway function together to maintain chromosomal stability.

Theoretically, the cGAS/STING pathway might affect chromosomal stability through actions during interphase that subsequently induce CIN during mitosis or through direct effects on mitotic progression. In terms of the first of these two possibilities, there is literature supporting the conclusion that in interphase cells, cGAS is capable of detecting dsDNA inside micronuclei with fragile envelopes and subsequently eliciting a downstream pathway that induces transcription of inflammatory cytokines and chemokines via the transcription factors IRF3 and NF-κB. The net effect of the operation of this pathway is to recruit cytotoxic T cells to the tumor microenvironment and promote apoptotic cell death, thereby indirectly decreasing the number of cells with CIN. It has also been reported that IRF3 can induce transcription of p53, resulting in upregulation of p21, which arrests cell-cycle progression^21,22^. Here, we clearly showed that IRF3-dependent downregulation of p21 is involved in cGAS depletion-induced micronuclei formation via precocious entry into mitosis (Figs. 6 and 7). In terms of possible direct effects of the cGAS/STING pathway during mitosis, the cGAS/STING pathway might affect microtubule stability or spindle assembly, given that TBK1 is known to interact with the centrosome proteins Cep170 and NuMA to regulate mitotic progression^19^. The cGAS/STING pathway may also be involved in various mitotic events, including cytokinesis, mitotic checkpoint function and mitotic cell death, as well as progression at prometaphase (Fig. S3). However, dissecting the direct role of the cGAS/STING pathway in mitotic progression through regulation of the G2/M transition independent of p21 will require additional and more precisely designed studies.

Various outcomes have been attributed to the downregulation of p21 during the interphase of cell-cycle progression. The first is that p21 downregulation can force mitotic entry of S phase-arrested cells, since p21 is no longer available to inhibit the formation of cyclin B1 and CDK1 complexes, resulting in premature mitotic entry that initiates before DNA synthesis is complete. This gives rise to abnormal mitotic phenotypes with dispersed chromosomes and disorganized bipolar spindle assembly. These mitotic cells will “slip through” this forced mitosis, leading to gross micronucleation and apoptotic cell death^25,27^. The second outcome follows from the fact that p21 is considered the sole regulator of the G2/M DNA damage checkpoint. When p21 is depleted, cells with DNA double-strand breaks in the preceding S phase can transit into the M phase without proper DDR because p21 can no longer inhibit the phosphorylation of CDK1 at threonine 161, which is necessary to enforce the G2 DNA damage checkpoint, leading to chromosome missegregation events and micronuclei formation^28–31^. This is consistent with our observations, given that p21 downregulation was able to enhance micronuclei formation and support precocious G2/M transition, giving rise to CIN. However, we could not detect an increase in γH2AX foci to prove our hypothesis that a decrease in p21 levels can result in inefficient DDR in the preceding interphase, leading to enhanced micronuclei formation in our system (Fig. S2).

There are two theoretically possible mechanisms by which a decrease in the activity of the cGAS/STING/TBK1/IRF3 pathway might downregulate p21. One is that the decrease in p21 levels is a consequence of transcriptional changes that would otherwise be controlled by the cGAS/STING pathway, which is depleted; this possibility is based on the fact that p21 levels are mainly regulated at the transcriptional level via various mechanisms. This mechanism predicts the possible involvement of IRF3 and/or NF-κB transcriptional activity. The second possible mechanism is that changes in p21 levels that occur after depletion of the cGAS–STING pathway are attributable to post-translational changes. The half-life of p21 in actively dividing cells has been reported to be 20–60 min^32^. Three E3 ubiquitin ligase complexes, SCF^Skp2^, CRL4^Cdt2^, and APC/C^Cdc20^, are involved in p21 degradation at specific stages of the cell cycle. Since APC/C^Cdc20^ is known to be involved in p21 degradation during G2 and M phases, it may be involved in p21 downregulation in these circumstances^33^. Moreover, there are two other reports suggesting that post-translational modification of p21 protein—acetylation and deubiquitylation mediated by Tip60 and USP11-deubuquitylase, respectively—regulates cell-cycle progression and DNA damage responses by increasing p21 expression levels^34,35^. These observations suggest the need for additional studies on the possible association between the cGAS/STING/TBK1/IRF3 axis and the post-translational modification of p21 protein. Ongoing efforts in our laboratory are focused on deciphering the precise mechanism underlying the cGAS/STING pathway-dependent regulation of p21 levels.

## Supplementary information

Supplementary Data
