# Supplementary material for: The cGAS/STING/TBK1/IRF3 innate immunity pathway maintains chromosomal stability through regulation of p21 levels
Source: Exp Mol Med. 2020 Apr 13;52(4):643–57. doi: 10.1038/s12276-020-0416-y (PMC7210884; doi:10.1038/s12276-020-0416-y)
Supplement: Supplementary file 1 — Supplementary Data [file 12276_2020_416_MOESM1_ESM.ppt]

## Slide 1
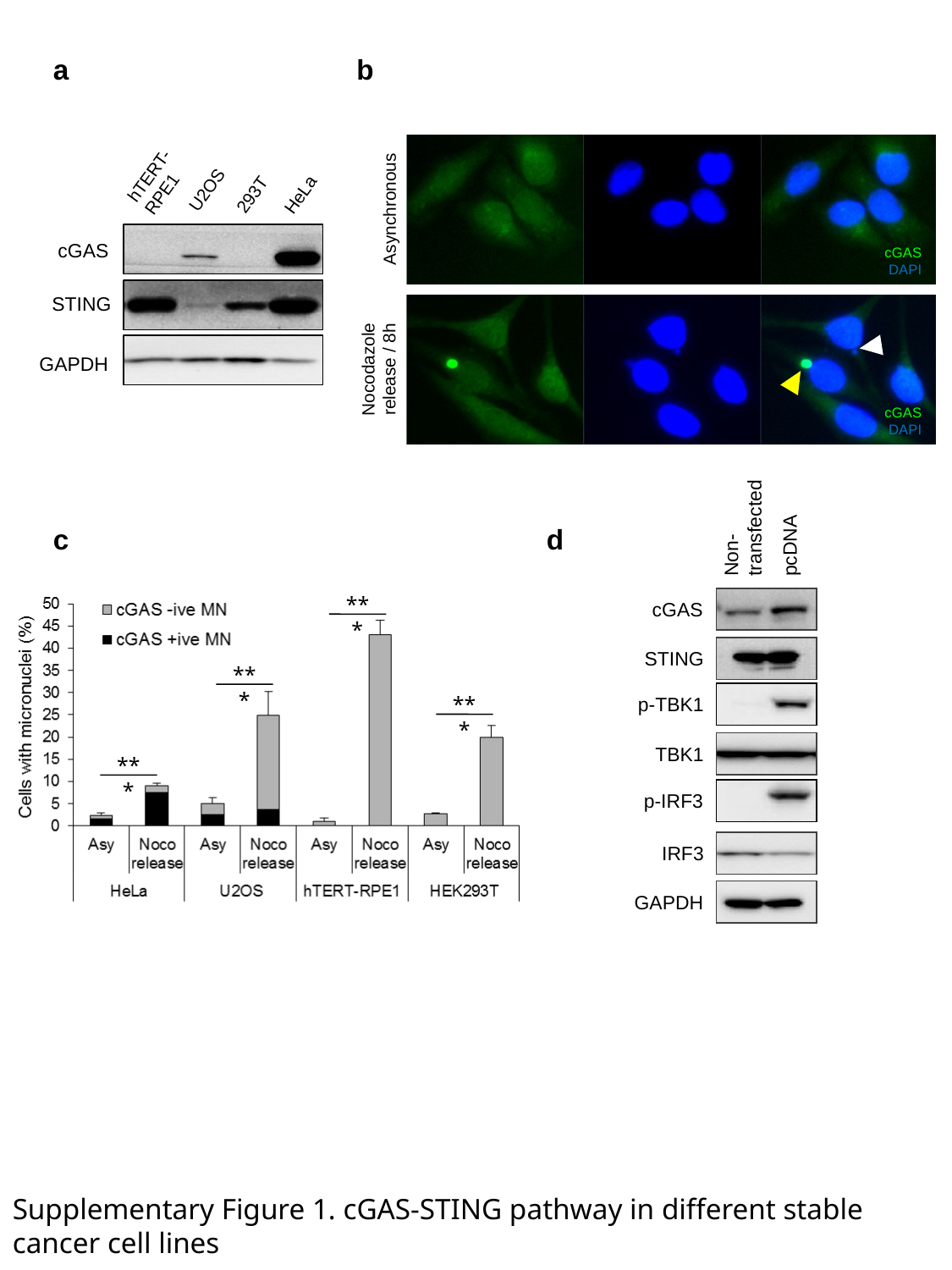

a
b
hTERT-
RPE1
U2OS
293T
HeLa
cGAS
STING
GAPDH
cGAS
DAPI
cGAS
DAPI
Asynchronous
Nocodazole
release / 8h
Non-
transfected
pcDNA
cGAS
STING
p-TBK1
TBK1
p-IRF3
IRF3
GAPDH
c
d
***
***
***
***
Supplementary Figure 1. cGAS-STING pathway in different stable cancer cell lines

## Slide 2
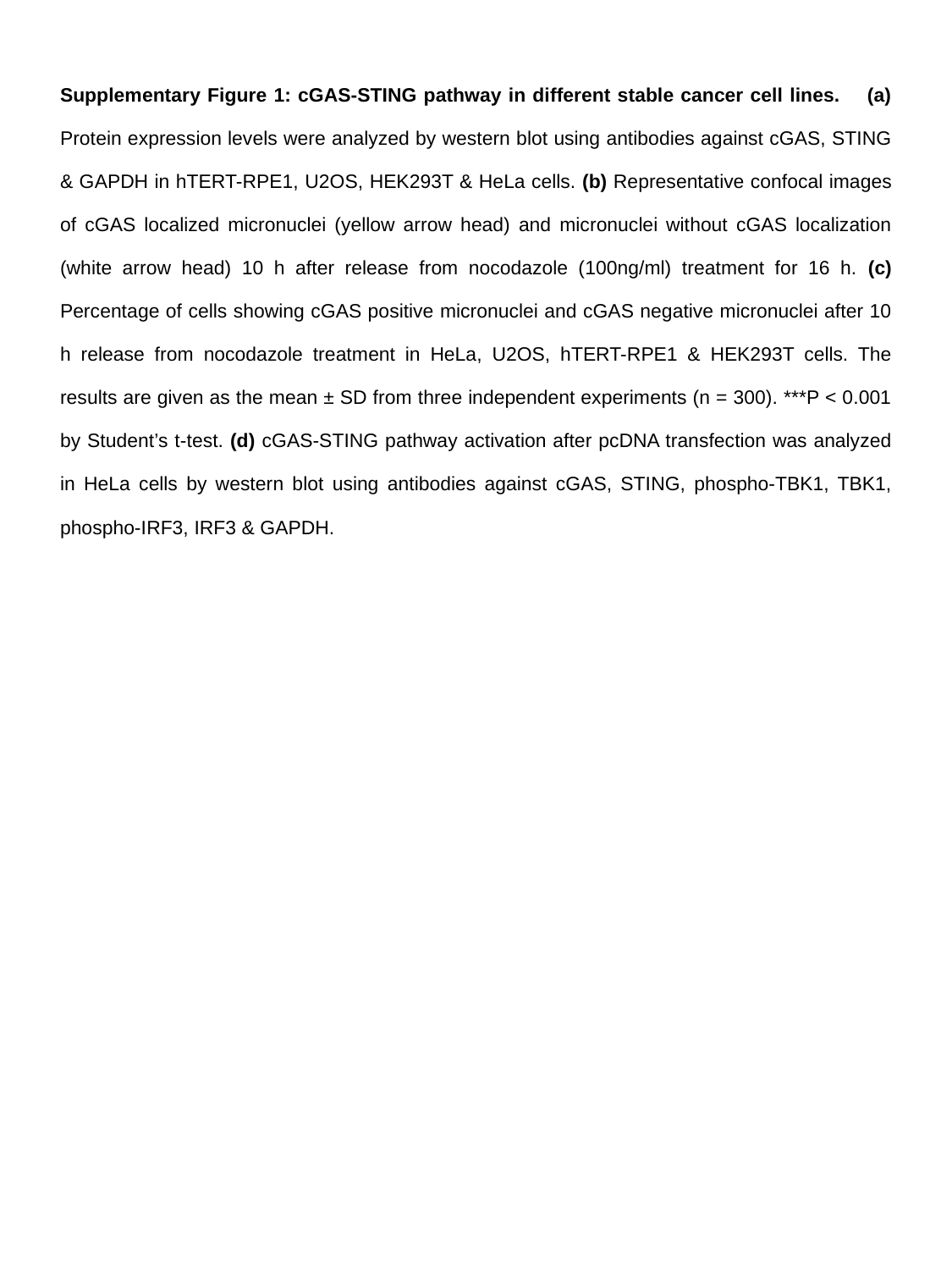

Supplementary Figure 1: cGAS-STING pathway in different stable cancer cell lines. (a) Protein expression levels were analyzed by western blot using antibodies against cGAS, STING & GAPDH in hTERT-RPE1, U2OS, HEK293T & HeLa cells. (b) Representative confocal images of cGAS localized micronuclei (yellow arrow head) and micronuclei without cGAS localization (white arrow head) 10 h after release from nocodazole (100ng/ml) treatment for 16 h. (c) Percentage of cells showing cGAS positive micronuclei and cGAS negative micronuclei after 10 h release from nocodazole treatment in HeLa, U2OS, hTERT-RPE1 & HEK293T cells. The results are given as the mean ± SD from three independent experiments (n = 300). ***P < 0.001 by Student’s t-test. (d) cGAS-STING pathway activation after pcDNA transfection was analyzed in HeLa cells by western blot using antibodies against cGAS, STING, phospho-TBK1, TBK1, phospho-IRF3, IRF3 & GAPDH.

## Slide 3
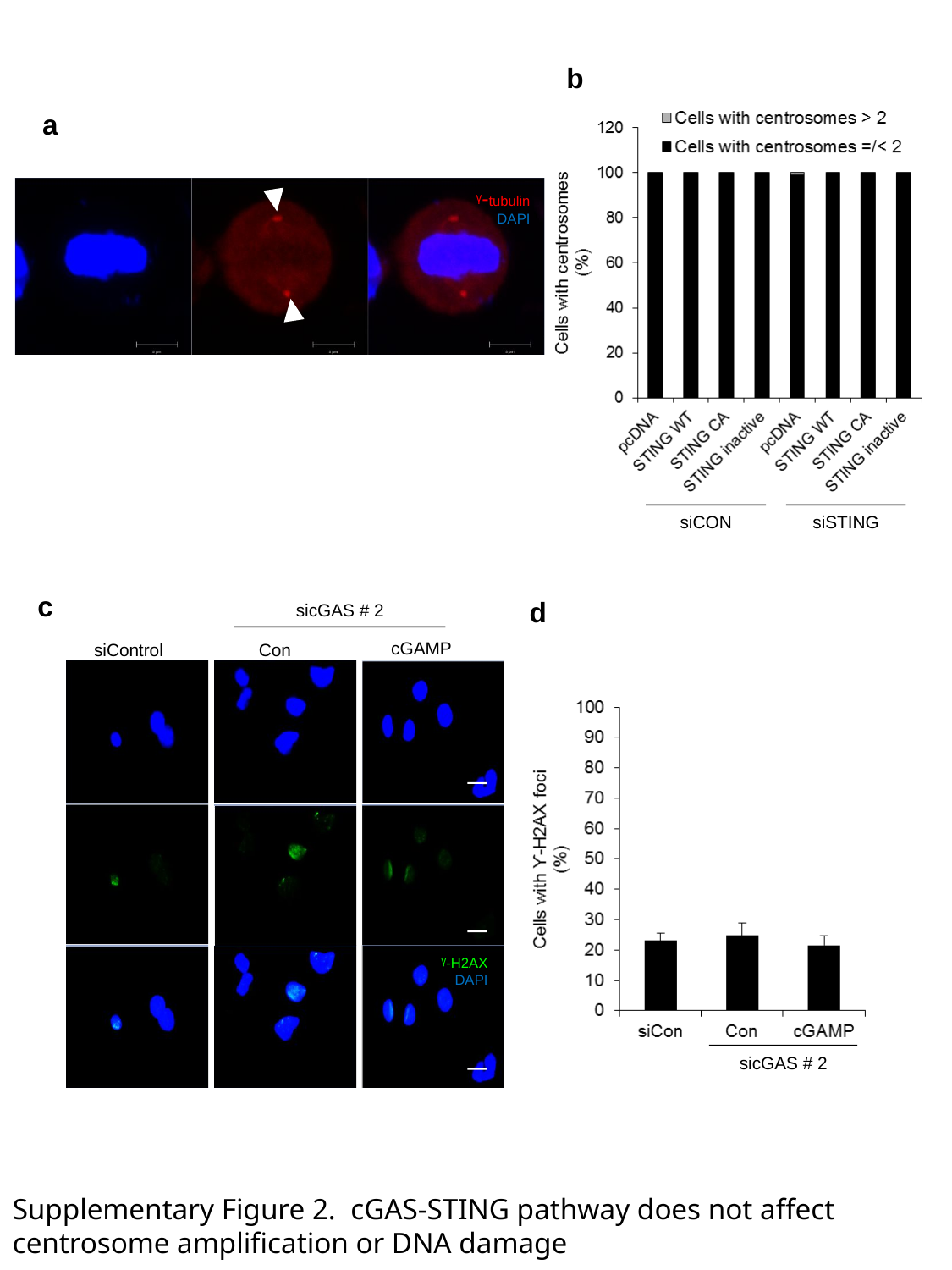

b
a
ᵞ-tubulin
DAPI
siCON
siSTING
c
sicGAS # 2
cGAMP
siControl
Con
ᵞ-H2AX
DAPI
d
sicGAS # 2
Supplementary Figure 2. cGAS-STING pathway does not affect centrosome amplification or DNA damage

## Slide 4
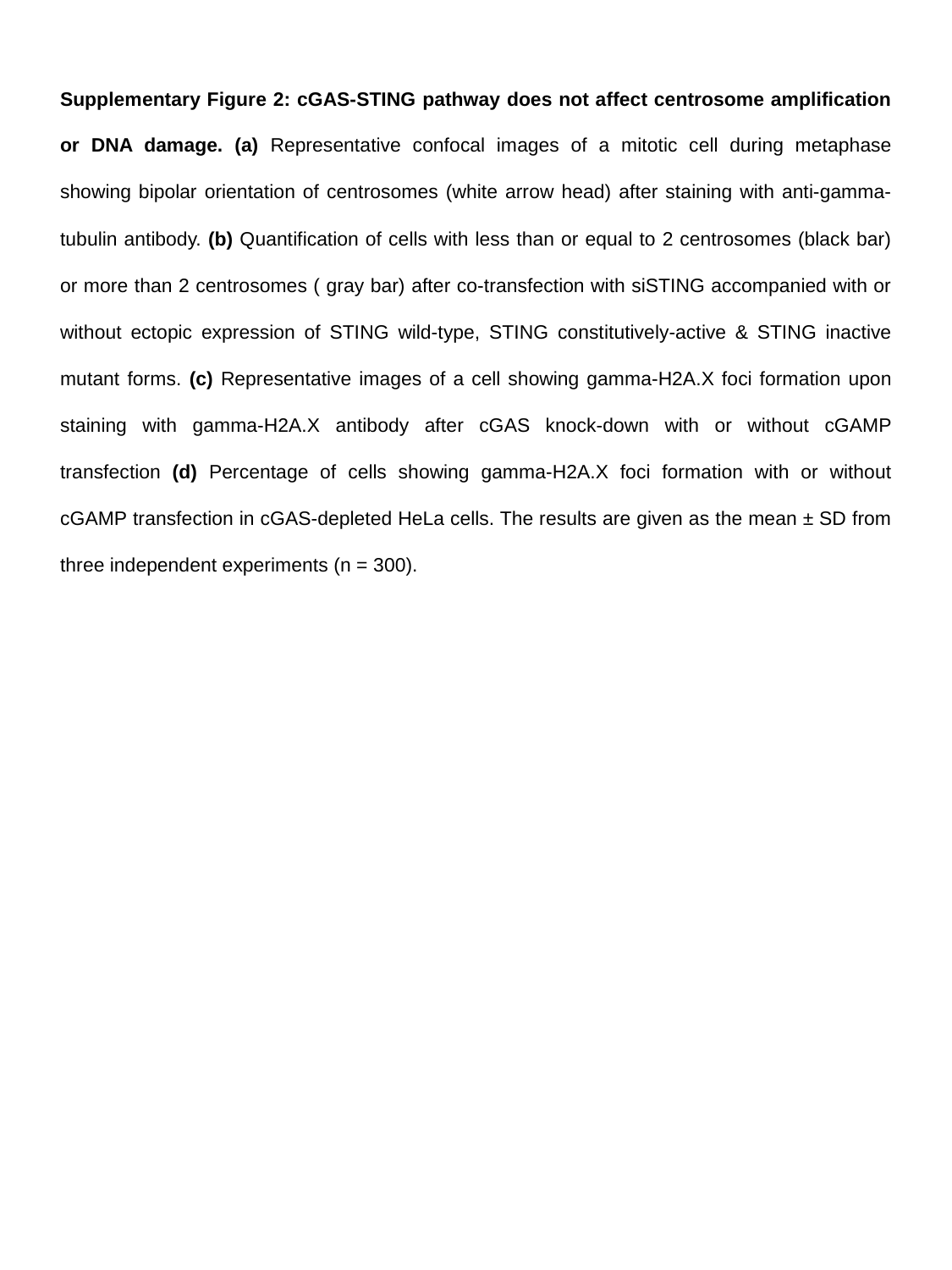

Supplementary Figure 2: cGAS-STING pathway does not affect centrosome amplification or DNA damage. (a) Representative confocal images of a mitotic cell during metaphase showing bipolar orientation of centrosomes (white arrow head) after staining with anti-gamma-tubulin antibody. (b) Quantification of cells with less than or equal to 2 centrosomes (black bar) or more than 2 centrosomes ( gray bar) after co-transfection with siSTING accompanied with or without ectopic expression of STING wild-type, STING constitutively-active & STING inactive mutant forms. (c) Representative images of a cell showing gamma-H2A.X foci formation upon staining with gamma-H2A.X antibody after cGAS knock-down with or without cGAMP transfection (d) Percentage of cells showing gamma-H2A.X foci formation with or without cGAMP transfection in cGAS-depleted HeLa cells. The results are given as the mean ± SD from three independent experiments (n = 300).

## Slide 5
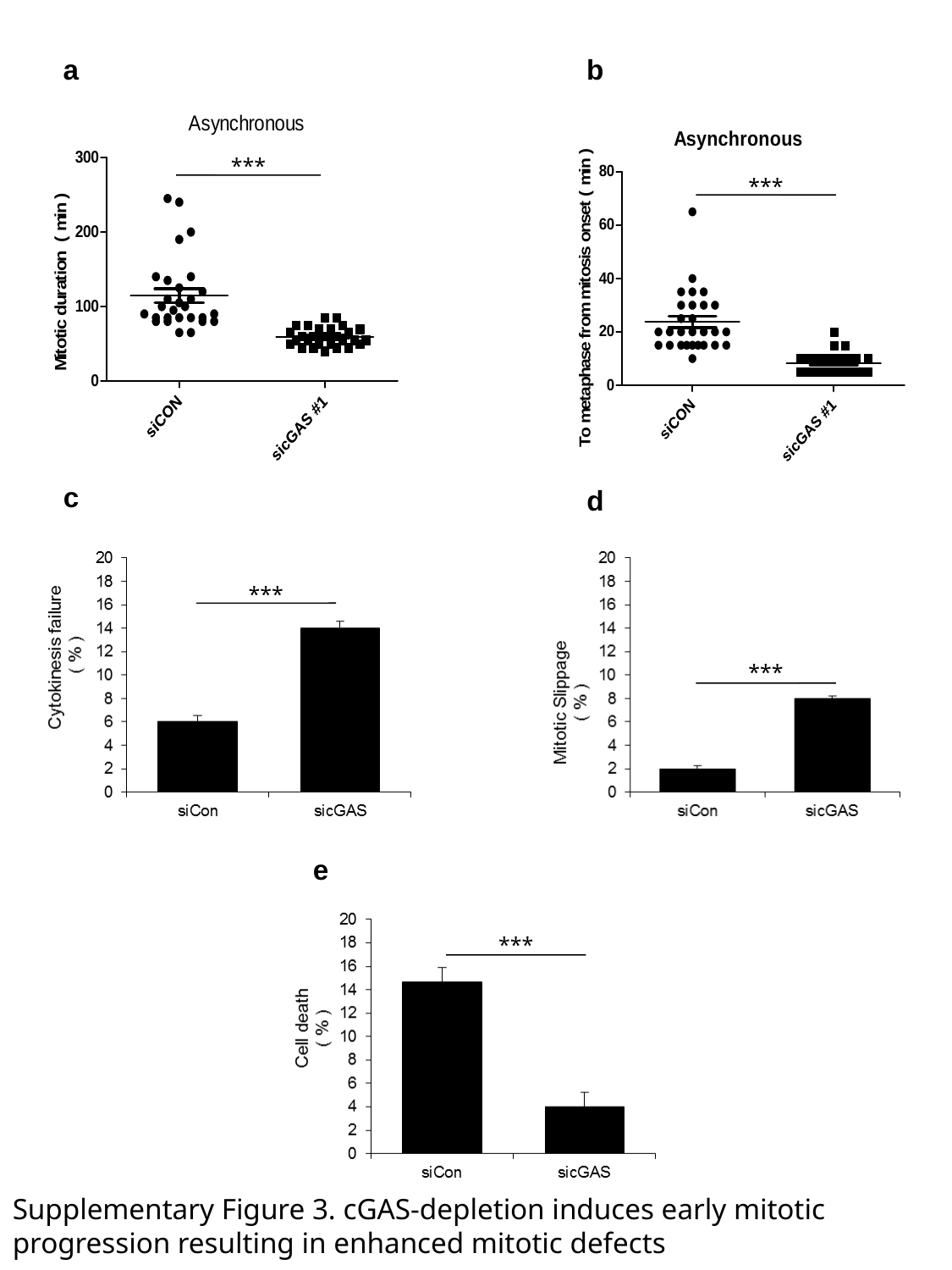

a
b
***
***
c
d
***
***
e
***
Supplementary Figure 3. cGAS-depletion induces early mitotic progression resulting in enhanced mitotic defects

## Slide 6
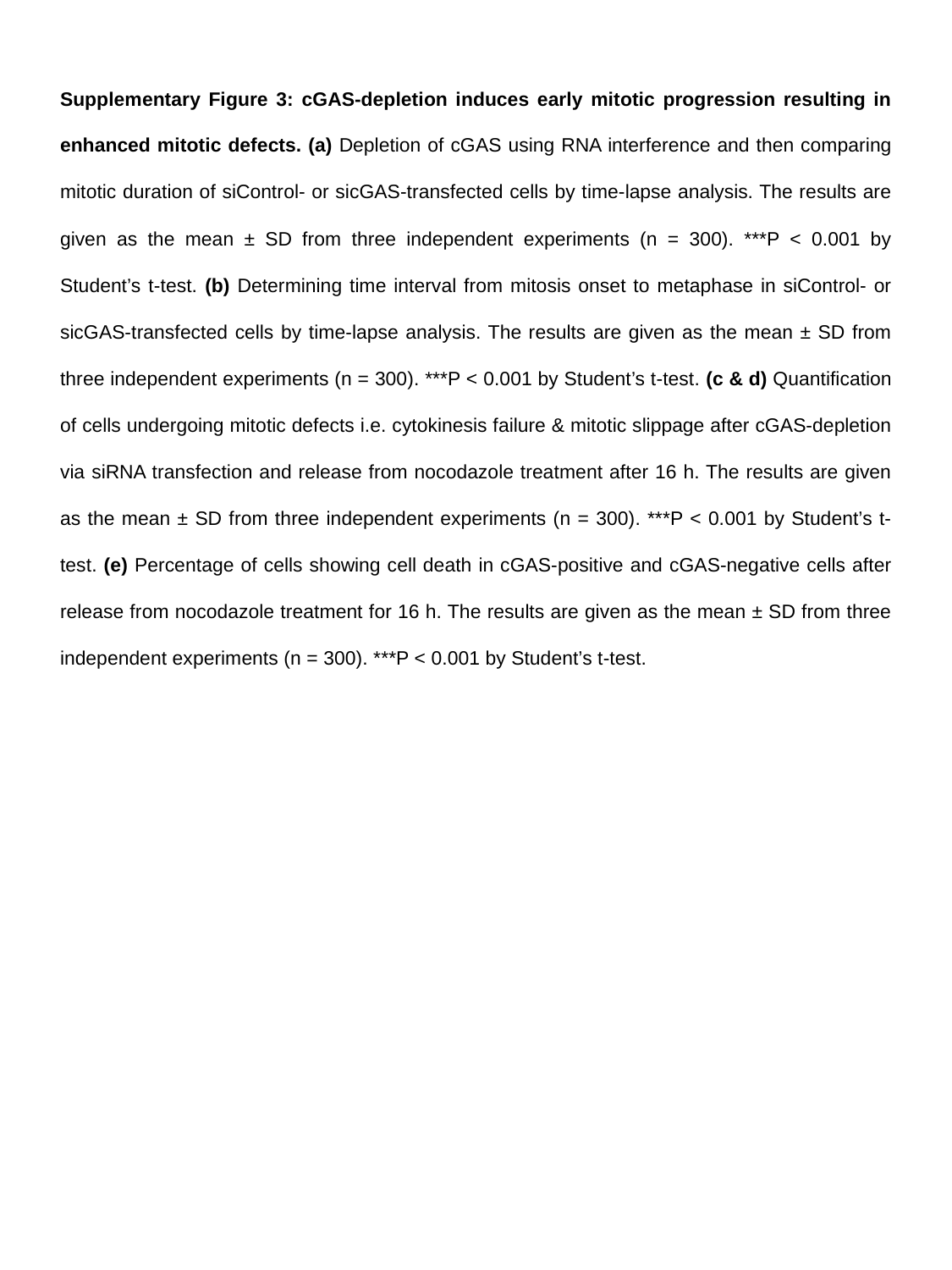

Supplementary Figure 3: cGAS-depletion induces early mitotic progression resulting in enhanced mitotic defects. (a) Depletion of cGAS using RNA interference and then comparing mitotic duration of siControl- or sicGAS-transfected cells by time-lapse analysis. The results are given as the mean ± SD from three independent experiments (n = 300). ***P < 0.001 by Student’s t-test. (b) Determining time interval from mitosis onset to metaphase in siControl- or sicGAS-transfected cells by time-lapse analysis. The results are given as the mean ± SD from three independent experiments (n = 300). ***P < 0.001 by Student’s t-test. (c & d) Quantification of cells undergoing mitotic defects i.e. cytokinesis failure & mitotic slippage after cGAS-depletion via siRNA transfection and release from nocodazole treatment after 16 h. The results are given as the mean ± SD from three independent experiments (n = 300). ***P < 0.001 by Student’s t-test. (e) Percentage of cells showing cell death in cGAS-positive and cGAS-negative cells after release from nocodazole treatment for 16 h. The results are given as the mean ± SD from three independent experiments (n = 300). ***P < 0.001 by Student’s t-test.
